# Intra-operative multi-site stimulation: Expanding methodology for cortical brain mapping of language functions

**DOI:** 10.1371/journal.pone.0180740

**Published:** 2017-07-10

**Authors:** Tal Gonen, Tomer Gazit, Akiva Korn, Adi Kirschner, Daniella Perry, Talma Hendler, Zvi Ram

**Affiliations:** 1 Department of Neurosurgery, Tel Aviv Medical Center, Tel-Aviv, Israel; 2 Tel Aviv Center for Brain Function, Tel Aviv Medical Center, Tel-Aviv, Israel; 3 School of Psychological Sciences, Tel-Aviv University, Tel-Aviv, Israel; 4 Sagol School for Neuroscience, Tel-Aviv University, Tel-Aviv, Israel; 5 Sackler School of Medicine, Tel-Aviv University, Tel-Aviv, Israel; Wadsworth Center, UNITED STATES

## Abstract

Direct cortical stimulation (DCS) is considered the gold-standard for functional cortical mapping during awake surgery for brain tumor resection. DCS is performed by stimulating one local cortical area at a time. We present a feasibility study using an intra-operative technique aimed at improving our ability to map brain functions which rely on activity in distributed cortical regions. Following standard DCS, Multi-Site Stimulation (MSS) was performed in 15 patients by applying simultaneous cortical stimulations at multiple locations. Language functioning was chosen as a case-cognitive domain due to its relatively well-known cortical organization. MSS, performed at sites that did not produce disruption when applied in a single stimulation point, revealed additional language dysfunction in 73% of the patients. Functional regions identified by this technique were presumed to be significant to language circuitry and were spared during surgery. No new neurological deficits were observed in any of the patients following surgery. Though the neuro-electrical effects of MSS need further investigation, this feasibility study may provide a first step towards sophistication of intra-operative cortical mapping.

## Introduction

Direct Cortical Stimulation (DCS) originated over half a century ago by Dr. Wilder Penfield, aiming to map the cortical surface in humans and as a result, minimize iatrogenic neurological deficits during surgery. The implementation of this technique allows for more radical and extensive tumor and epileptic foci resections while minimizing the risk of postoperative neurological deficits [1,2]. Furthermore, this approach provided the first detailed functional maps of the human brain (e.g. the homunculus)[3] and was a cornerstone for subsequent neuroscience research. Ojemann et al. (1979) have expanded functional mapping to cognitive functions, identifying, for example, subdivisions within previously known language regions [4]. Over the last few decades, DCS has become a widely used technique for intra-operative mapping during both awake and anesthetized surgery in tumor and epilepsy patients[5], and has been described as superior to pre-operative mapping using functional MRI[6]. Nevertheless, in spite of its advantages, DCS may fail to prevent postoperative cognitive decline in some patients (from 6 to 20%[7,8]) or result in suboptimal brain tumor resection[9].

The electrical current distribution of standard bipolar DCS has been shown to be a local half sphere. For example with a 10 mA bipolar stimulation (with an electrode diameter of 3mm), Nathan et al. (1993)[10] have shown that the peak current density occurs in the region immediately beneath the bipolar electrodes (0.05 A/cm^2^) and it declines rapidly to 0.02 A/cm^2^, at a radius of 0.5 cm. However, both electrophysiological and neuroimaging studies have shown that cognitive functions are represented by neural activations in a variety of spatial organizations depending on cortical and subcortical anatomy and functionality[11]. This may be manifested at non-spherical organization or distributed cortical activations representing higher brain functions, such as language, memory and emotions. In such cases, an effective spherical stimulation of cortex may be suboptimal since low current stimulation may not elicit a cognitive response (as they do not cover the extent of the relevant cortical tissue) while high current may elicit epileptogenic activity, or result in suboptimal resection. A method for stimulating non-spherical and even non-continuous cortical areas may be advantageous in defining the extent of cortical surface necessary for specific brain functions. One possibility to overcome this limitation is to simultaneously stimulate multiple cortical sites, thereby effecting non-spherically organized cortical surfaces.

In this pilot study, we aimed to evaluate whether simultaneous stimulation of several cortical sites may result in cognitive effects not observed with the standard single site DCS. Using language mapping as a case study we evaluated different aspects of language production and comprehension while applying simultaneous Multi Site Stimulation (MSS) in patients who underwent awake craniotomy for removal of a brain tumor.

The abundant cohort of language related imaging studies have created a distributed and complex map of cortical regions involved in language processing[12]. However, due to the indirect nature of functional imaging, these studies lack the ability to indicate the criticality of cortical structures in specific functions. To date, evidence on neural structures criticality has come from either lesion studies or single site DCS. Thus, despite the distributed network involved in language processing, it is still possible that the different language functions rely on specific, local and critical areas, or critical “hot-spots” for which single site, focal stimulation would be the optimal mapping technique. However, if language functioning is also dependent on interplay between more distributed and even separated cortical tissues, we would suspect that MSS could be beneficial in mapping such language processing. Our initial hypothesis was that MSS will enable the detection of language function disruptions not observed using single site stimulation.

## Methods

### Patients

Data were prospectively collected from non-consecutive patients who underwent awake craniotomy for resection of intra-axial tumors with mapping of language functions at the Tel-Aviv Medical Center between November 2012 and October 2013.

Exclusion criteria were the presence of severe preoperative language deficit that prevented comprehensive testing and mapping, or occurrence of seizures during standard cortical mapping. The study was approved by the Tel Aviv Medical Center Institutional Review Board (IRB approval number TLV-0293-13), and carried out in accordance with these approved guidelines. Written informed consent was obtained from all subjects.

15 patients who underwent awake craniotomy for tumor removal met the inclusion criteria and participated in this study (9 males, 1 left handed; age 42.86±14.2). Nine patients harbored high grade glioma, five low grade gliomas and one cavernoma. Six lesions were located in the temporal lobe, three in the frontal lobe and six in the insula or fronto-insular region. All lesions were located in the left hemisphere.

### Clinical characteristics

The patients’ demographic, clinical, perioperative and hospitalization data were recorded. The Karnofsky Performance Scale (KPS) was used to assess preoperative general functional status. The extent of tumor resection (EOR) was established by a postoperative MRI scan performed within 48 hours of surgery and categorized as follows: gross total resection ([GTR] >95%) if no residual tumor enhancement was detected and subtotal resection ([STR] >90%) if slight residual tumor enhancement was detected. FLAIR MRI scan was used to asses EOR in Low Grade Glioma. Tumor pathology was determined by a neuro-pathologist based on the World Health Organization (WHO) criteria [13]. Perioperative mortality was recorded within 30 days after surgery. Postoperative neurological outcome was assessed within 7 days from surgery. Length of hospital stay (LOS) was collected from the hospital records. See Table 1 for full clinical and demographic characteristics.

**Table 1:** Clinical and demographic data.

Patient #	Age (years)	Gender	Dominant hand	KPS	Anatomical location	Pathology	Extent of resection	Pre-operative language function	Post-operative language function
**1**	60	M	Right	100	Frontal	Oligo—II	GTR	Intact	Intact
**2**	33	M	Right	100	Temporal	Astro—III	GTR	Intact	Intact
**3**	38	M	Right	100	Temporal	GBM	STR	Mild sensory dysphasia	Improve
**4**	31	F	Right	80	Frontal	GBM	STR	Mild motor dysphasia	Improve
**5**	36	M	Right	100	Insular	GBM	STR	Intact	Intact
**6**	40	M	Right	100	Insular	Oligo—II	GTR	Intact	Intact
**7**	37	F	Right	90	Insular	Astro—II	STR	Mild motor dysphasia	Improve
**8**	33	F	Right	100	Insular	Astro—II	GTR	Intact	Intact
**9**	27	F	Right	90	Temporal	GBM	GTR	Mild sensory dysphasia	Improve
**10**	23	M	Left	100	Temporal	ICH	GTR	Intact	Intact
**11**	66	M	Right	100	Temporal	GBM	STR	Sensory dysphasia	Improve
**12**	54	F	Right	100	Frontal	GBM	GTR	Sensory dysphasia	Improve
**13**	62	F	Right	90	Frontal	GBM	GTR	Motor dysphasia	Improve
**14**	63	M	Right	90	Temporal	GBM	STR	Motor dysphasia	Improve
**15**	40	M	Right	100	Fronto-insular	Oligo—II	STR	Intact	Intact

Abbreviations: M = male; F = female; KPS = Karnofski performance scale; Oligo = oligodendroglioma; Astro = astrocytoma; the number (II, III) stands for WHO grade; GBM = Glioblastoma; ICH = intra cerebral hemorrhage; GTR = gross total resection (no residual tumor enhancement was detected in post-operative MRI (>95%)); STR = Sub Total resection (slight residual tumor enhancement was detected (>90%)).

### Preoperative imaging

Anatomical MRI scans were performed for all patients prior to surgery using a 3-Tesla GE scanner (signa ECITE). Anatomical 3D sequence spoiled gradient echo (SPGR) sequence was obtained with high-resolution 1-mm slice thickness for all patients. SPGR sequence was performed twice; before and after injection of Gadolinium contrast agent. The subtraction map of post- minus pre- injection SPGR scans was used for the creation of anatomical vascular maps, which were used to assist in registration of intraoperative photography with preoperative MRI. For patients with suspected low-grade tumors, a high-resolution fluid-attenuated inversion recovery (FLAIR) sequence was also conducted for intraoperative navigation. fMRI was obtained for 14 patients to establish hemispherical language lateralization using a single-shot echo planar T2*-weighted sequence (TR/TE = 3000/35msec, flip angle 90°, FOV = 200mm, 40 slices with 3mm thickness, no gap, matrix 96×96). Subjects performed four tasks: a visual and auditory verb generation task as described in Gazit et al. 2016 [14], and a visual and auditory definition task in which subjects were asked to covertly answer simple questions such as "what protects against rain?". All four tasks were block design and lasted 3:24, 3:03, 3:12 and 3:24 minutes respectively. For all patients, including left handed ones, language was lateralized to the left hemisphere.

### Functional preoperative preparation

The standard pre-operative evaluation for patients considered candidates for awake craniotomy was applied (for detailed description of standard protocol see[15]). A functional evaluation was carried out 1–2 days prior to surgery to establish each patient’s baseline level of functioning.

#### Language functions testing

Three tasks were generally used for language mapping during cortical stimulations: Naming, Verb Generation and Comprehension/ semantic retrieval.

1. Naming—a commonly used task[15,16] designed for mapping of pronunciation, visual object recognition and naming. Patients were presented with pictures of concrete objects (e.g. a car, a banana) from several semantic categories (e.g. food, clothing items) and were requested to name them. Patients were asked to start each answer with the words "This is a ____ (name—e.g. "car")", in order to separate pronunciation from the naming.
2. Verb Generation (VG)–in this task patients were requested to retrieve an appropriate verb describing an action related to a presented noun. As different objects vary in the number of appropriate verb complements (e.g. you generally eat a "banana" but you can live in, visit, or tour a "palace"), the test included objects of several difficulty levels and thus enabled sensitive measurement of patients' deficit in the task. Visually presented pictures were used for combining a Naming and VG task and patients were asked to respond to each picture according to a fixed pattern: "This is a ______ (name—e.g. "ball") and you ______ (verb—e.g. "play") with it", in order to dissociate the performance of each task. This combination allowed for better classification of performance, dissociation between functional disturbances and shortened the duration of the cortical stimulations.
3. Comprehension and semantic retrieval—in this task patients were asked to retrieve nouns in response to auditory presented definitions (i.e. definitions were read to the patients). The test included definitions of concrete objects (e.g. "a yellow sour fruit"–a lemon), as well as of more abstract nouns (e.g. "teaches children in class"–a teacher), and was used in order to map semantic retrieval and comprehension. When a stimulation caused for comprehension disturbance (i.e. patient could not retrieve any answer to a definition; not in case of paraphasia, hesitation etc.), simple instructions were used during stimulation of the same site to determine whether this was a comprehension (word deafness) or a retrieval disturbance.

According to our experience this language-battery provides a comprehensive account of language functions including dissociation between phonemic and semantic deficits, anomia vs. retrieval difficulties etc. This same battery was repeated intra-operatively during functional mapping and monitoring (see below). A short post-operative evaluation was conducted within a week from surgery by the clinical team.

### Intraoperative management and standard functional mapping

Intraoperative anesthetic management was in line with the standard protocol applied during awake craniotomy in our facility (for details see[15]). Importantly, all sedatives and analgesics were stopped after head fixation in order to ensure patients’ focused attention throughout cognitive mapping and monitoring.

Intraoperative functional baseline was established at least 30 minutes following termination of sedative administration. This baseline was conducted after the patients’ head was fixated but before skin incision in order to control for stress-related functional decline, as well as to establish within-subject test-retest reliability; so that only items answered correctly in both baseline sessions were used for cortical mapping.

Electrocorticographic (ECOG) based after-discharges were monitored using an 8 contact subdural cortical strip electrode (Ad Tech Medical Instrumentation Corp., Racine, WI, USA) connected to an intraoperative neurophysiological workstation (NIM Eclipse, Medtronic, Minneapolis, MN USA) analyzed by an experienced clinical neurophysiologist. If after-discharges were detected, the epileptogenic activity was abrogated with irrigation of the cortical surface with ice water. Direct cortical 50 Hz bipolar (biphasic pulses, 500-msec pulse width, and 2–3-second train duration) stimulation was performed for cortical mapping of language functions[17] using a handheld Ojemann Cortical Stimulator with two ball tips (Radionics Inc., Burlington, MA) with 0.5cm spacing between electrode tips. The cortical surface was stimulated in 2mA intensity increments, ranging from 4-10mA or until a functional response was elicited (mean±SD of highest current intensity applied was 6.53±1.59mA). During stimulation different aspects of language production and comprehension were tested, using only successful items from both baseline evaluation sessions. Performance was evaluated based on response time and accuracy as compared to patient’s intraoperative baseline, with respect to specific characteristics of the patient’s language performance as established in both pre-and intra-operative baseline. This ensured that mistakes or disturbances which occurred during stimulations were the result of interruption to a functional region and not an incidental one. Functional disruption was determined if the same effect was consistently replicated three times with no evidence of seizure activity. Effects of stimulation and current intensity on behavior and performance (e.g., speech arrest, anomia) were documented and captured using Sonowand navigation system (SonoWand, Mison). Following cortical stimulation and throughout the resection language functions were monitored using free speech and conversation with the observer.

### Multi-site cortical stimulation (MSS)

Following single-site stimulations (SSS) multi-site stimulation (MSS) was performed. 50Hz, 3-second bipolar stimulation was undertaken at two cortical sites simultaneously using 4 paired contacts of the 8-contact cortical electrode (AdTech) with the remaining 4 used for ECOG monitoring. The contacts within a pair were always situated adjacent to one another along the strip electrode, separated by a distance of 1 cm, contact center-to contact center. The two pairs of stimulation were carried out using two separate and isolated stimulation ports on the surgical evoked response unit (Medtronic, NIM Eclipse 32 channel neurologic workstation).

The intensity ranged from 4-8mA, depending upon the predetermined intensity to elicit a SSS cognitive or motor response (to reduce the probability for after discharges and due to time limitations 10mA stimulation was not attempted for MSS). When no single-site effect was observed, MSS were applied using current intensity of 6mA, which from our experience is sufficient in most cases to elicit a functional response on the one hand, while minimizing the risk for intraoperative seizures on the other. The strip electrode was placed on a silent cortex, (where no functional disruptions occurred during SSS). Each pair of electrodes was first stimulated separately to ensure it was indeed silent cortex and no functional disruption occurred upon a single bipolar stimulation. In the two simultaneous biphasic bipolar stimulations within the ECOG strip, the onset-anode and onset-cathode electrode within each pair was randomly selected. After testing for MSS of two sites using 4 paired contacts of the electrode, as well as the maximal technical ability of the intraoperative neurophysiologic monitoring (IOM) unit to produce simultaneous stimulation, a third site was added using the Ojemann bipolar stimulator at the same current intensity as used with the other two stimulation sites. Additional functional disruptions (if occurred consistently for three times), were documented. The ECoG strip and Ojemann stimulator were manually operated by two separate personnel and thus were not phase locked.

To avoid false positive mapping as well as intraoperative seizures, ECOG was continuously monitored within the area of SSS and MSS during stimulation using at least 4 channels. In the event of recognized stimulation-related epileptogenic activity, sessions resulting in cognitive disruption were not considered as true positives. Patients who experienced seizures or consistent stimulation-evoked epileptogenic ECOG activity during single-site stimulation were not tested with multisite stimulation to mitigate the occurrence of additional seizures or false positive mapping.

## Results

### Standard—Single-site stimulations (SSS)

Standard, single-site DCS (SSS) with a 50Hz bipolar stimulation (Ojemann Cortical Stimulator; Radionics Inc., Burlington, MA; see Methods for stimulation details) was performed for initial mapping of language function using several language tasks (e.g. object naming, sentence comprehension; for details see Methods). A functional evaluation was carried out before surgery to establish each patient’s baseline level of functioning, and the same tests were repeated intra-operatively during functional mapping. The cortical surface was stimulated in 2mA intensity increments, ranging from 4-10mA or until a functional response was elicited. Functional disruption was determined only if the same effect was consistently elicited for three times with no evidence of seizure activity (see Methods).

Speech arrest, indicative of primary production area (“Broca’s area”) was elicited in eight patients (in the inferior frontal gyrus [IFG]). Word deafness, indicating primary comprehension region (“Wernicke’s area”) was elicited in five patients (in the superior or middle temporal gyri [STG and MTG respectively]). Other language dysfunctions indicating secondary language regions, such as semantic, phonological or initiation disturbance were elicited in ten patients in various locations (frontal, temporal and parietal lobes). In three patients SSS did not induce any language dysfunction. Table 2 provides detailed description of all functional effects of SSS.

**Table 2:** Single-, two- and three- site stimulation functional effects.

patient #	Tumor location*	SSS	MSS—2 sites		MSS—3 sites	
			effect^Φ^	fMRI^£^	effect^Φ^	fMRI^£^
**1**	Frontal	None	Anomia	NA	Phonologic	NA
**2**	Temporal	Anomia, hesitation	Phonologic	Yes	None	NA
**3**	Temporal	Speech arrest, hesitation	Auditory hallucinations	Yes	Comprehension	No
**4**	Frontal	Hesitation, anomia	Slowness	NA	Discarded^§^	NA
**5**	Insular	Speech arrest, comprehension, anomia, VG†	None	Yes	None	NA
**6**	Insular	Speech arrest, comprehension, anomia, retrieval, semantic, phonemic	Hesitation	Yes	None	NA
**7**	Insular	Speech arrest, comprehension, phonologic	None	No	Discarded^§^	NA
**8**	Insular	Speech arrest, comprehension	Syntax	Yes	VG	Yes
**9**	Temporal	None	None	NA	None	NA
**10**	Temporal	Semantic	Retrieval /hesitation	No	Semantic	No
**11**	Temporal	Comprehension, semantic	Anomia	NA	Discarded^§^	NA
**12**	Frontal	None	Hesitation	NA	VG and hesitation	NA
**13**	Frontal	Speech arrest	Speech arrest	Yes	None	NA
**14**^¥^	Temporal	Speech arrest, semantic, phonologic	Phonologic Hesitation	No	None	NA
**15**	Fronto-Insular	Speech arrest, anomia	None	Yes	Phonologic	Yes

* All tumors were left sided;

^**Φ**^ For two site stimulations, all the effects were always additional to the effects found in SSS because we only evaluated MSS on silent SSS cortex. For three site stimulation, only additional effects (over those found with two site stimulations) are reported.

^†^ VG = verb generation disturbance;

^**¥**^ in this patient 2-site stimulation was performed using two Ojemann stimulators and two different effects were caused in two different locations.

^§^ Discarded due epileptogenic activity.

^**£**^NA = Not Applicable. Yes represents a convergence of at least one of the MSS sites with at least one of the four fMRI language tasks.

### Multi-site simultaneous stimulations (MSS)

#### Two-site simultaneous stimulations

**Fig 1:**
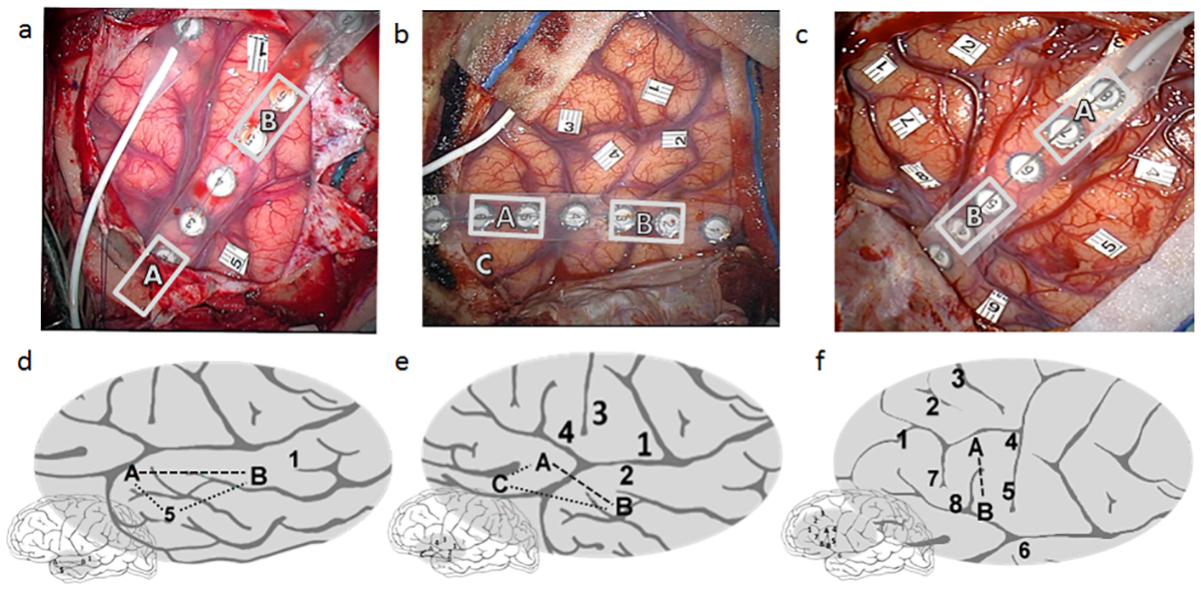
Examples of single and multi-site effects. One case example of a multi-site temporal effect (a,d—patient 10), one case example of a cross-lobal multi-site effect (fronto-temporal; b,e—patient 8) and one case example of a multi-site frontal effect (c,f—patient 6). (a-c) intra-operative photography, (d-f) stimulation location on scheme (effects were aligned using both angio and navigation system photographs). (a) Patient 10: single site DCS found a single word deafness effect (1). A cortical strip was placed on the superior temporal gyrus, and a two-site stimulation retrieval disturbance effect was found (A-B). An Ojemann stimulator was simultaneously applied on location 5, causing a three site semantic paraphasia effect. (b) Patient 8: single site DCS found a speech arrest effect (4), a word deafness effect (2) and two motor effects (1,3). A cortical strip was placed crossing the sylvian fissure, and a two-site stimulation syntax disturbance effect was found using a frontal site and a temporal site.). An Ojemann stimulator was simultaneously applied on location C, causing a three site verb-generation effect. (c) Patient 6: single site DCS found a speech arrest effect (5), five semantic effects (1,2,3,7,8) and a word deafness effect (6). A cortical strip was placed upon the inferior and middle frontal gyri and a two-site stimulation phonological effect was found. Dashed lines represent 2-site and 3-site MSS affects.

**Fig 2:**
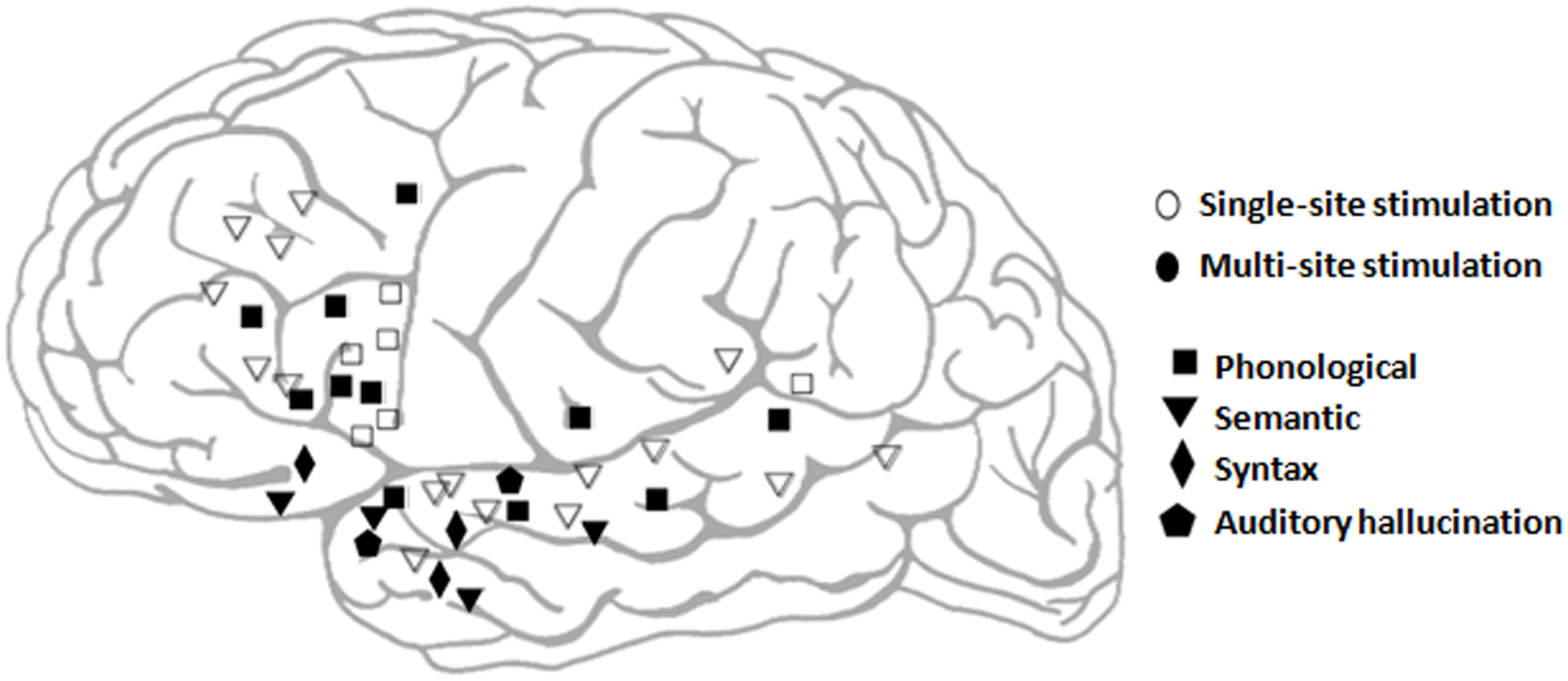
Schematic localization and classification of functional effects. The locations of stimulation sites (both single and multi) causing functional effects is plotted on a scheme of the left hemisphere for 7 of the 11 patients with multi-site effects (for the remaining four patients: 1,4,11 and 12, intra-operative imaging was not sufficient to map stimulation locations). Single sites (SSS) are marked with unfilled shapes, multi-site (MSS) are marked with filled shapes. Language disturbances are classified to phonological (square), semantic (triangle), syntactic (diamond) and auditory hallucinations (pentagon).

Following SSS, MSS was performed by simultaneously stimulating two pairs of an 8-contact cortical electrode strip (AdTech, Medical Instrumentation Corp., Racine, WI, USA). The strip electrode was placed on a silent cortex, (where no functional disruptions occurred during SSS). The remaining 4 strip contacts were used for ECOG monitoring. Fig 1 displays examples for MSS effects in cases of frontal, temporal and fronto-temporal craniotomies. In the event of recognized stimulation-related epileptogenic activity sessions were excluded (see Methods). In one patient (patient 14), multi-site stimulation was performed using two Ojemann stimulators simultaneously for technical reasons. In 10 of the 15 patients, MSS of two sites caused additional language dysfunctions that were not elicited using SSS (marked if occurred consistently three times, Table 2). Since we only performed two-site stimulation in one location pair per patient, this represents ten successful attempts out of 15. A binomial test found that the probability of obtaining at least ten successes from 15 random attempts (each with 0.05 probability for success) is less that 10^−6^. During two-site simultaneous stimulation, five patients exhibited production-related disturbances (speech arrest, phonological paraphasia, hesitation, and stutter) along the premotor cortex (Brodmann Area 6) and the IFG, as well as along the STG (Fig 2, Table 2). In three patients semantic-related effects occurred during two site stimulation (anomia, retrieval), located mostly at the anterior temporal lobe (superior and middle gyri) (Fig 2, Table 2). One patient exhibited syntax errors while stimulating two simultaneous sites (one in the IFG and one in anterior superior temporal sulcus [STS], Fig 1b and 1e); and one had experienced linguistic auditory hallucinations (during stimulation of two sites along the anterior STG). In three patients, during three site stimulation, ECOG epileptogenic activity was observed and accordingly data was discarded as non-reliable (data for 2-site stimulation is presented). Two patients did not experience any language dysfunctions with two-site stimulation. Data of dysfunctional effects for all patients is presented in Table 2, while summary of effects localization from eight of the MSS cases is shown in Fig 2 (for the remaining patients intra-operative imaging was insufficient to perform registration and localization).

#### Three-site simultaneous stimulations

After testing for MSS of two sites, a third site was added using the bipolar Ojemann stimulator at the same current intensity as used with the other two stimulation sites. Additional functional disruptions (if occurred consistently for three times), were documented (see Methods). Three-site MSS caused for additional language effects in eight patients. Production-related effects (phonological paraphasias, hesitation) which occurred in frontal regions (IFG, MFG; Fig 2) were elicited in five patients, and in three patients semantic-related effects (word deafness, semantic paraphasias) occurred, all located in the temporal lobe (STG, MTG; Fig 2). ECOG epileptogenic activity was observed in two patients and accordingly the relevant data was discarded. In five patients no additional dysfunction occurred by three-site simultaneous stimulation. The probability to obtain these results by chance could not be computed since in the case of three site stimulation, more than one location for the Ojemann stimulator was evaluated and only successful attempts were documented. Data of MSS effects for all patients is presented in Table 2.

#### Convergence with fMRI activations

For the ten patients (eight with positive MSS and two with negative MSS results) for whom we could localize the stimulation sites on the preoperative MRI, we evaluated whether MSS sites were localized on fMRI activations (T-maps). Thresholds for fMRI mapping were chosen per patients and task on clinical basis preoperatively (0.00001≤p≤0.01, 50≤cluster size≤100). A site was marked as converging with fMRI activation if the activation map of at least one of the four tasks reached less than 3mm from that site. Of the seven patients with a positive two-site MSS we found convergence with both MSS sites in two patients (patients 2 and 6), with one site in three patients (patients 3,8 and 13) and with none of the sites in two patients (10 and 14). Of the two patients with negative two-site MSS we found convergence with fMRI for one (patient 5). Of the three patients with a positive three-site MSS effect which could be localized (patients 3, 10 and 15), only patient 15 showed convergence of the location of the third stimulation site (Ojemann stimulator) with fMRI (S1 Fig).

### Surgical outcome

Eloquent cortex (all functional locations) identified in SSS and MSS was spared (in cases where MSS effect was found, the whole area between stimulation sites was spared) and the language functions of all patients were monitored throughout resection. A post-operative functional evaluation was conducted within a week from surgery. None of the patients experienced a new neurological deficit following surgery. Moreover, in six of the 10 patients who had a pre-operative language deficit and experienced dysfunction caused by MSS, post-operative language functions have improved, as assessed by the clinical team. Gross Total Resection (GTR, >95% using postoperative MRI) was achieved in eight patients and Sub-Total Resection (STR, >90%) in seven patients.

## Discussion

In this pilot study we demonstrate the feasibility to expand the state of the art intra-operative brain mapping technique. We showed that MSS allowed us to identify functional nodes that were not elicited with single-site, standard DCS. MSS of two- and three- sites revealed additive dysfunctions in 73% of the patients (11/15 patients), including two patients who did not experience any dysfunctions during SSS. Since each pair of electrodes was first stimulated separately and no functional disruption occurred upon single bipolar stimulation, the shift from Ojemann stimulator to the use of the ECoG strips could not explain the functional effects caused by MSS. Functional effects caused by MSS were not elicited by a simple current increase at specific locations since, across patients, MSS was performed with lower stimulation intensities to a specific region, compared to the SSS stimulation intensities (which in some cases did not elicit functional disruption up to 10mA). Thus, our original hypothesis that simultaneous stimulations of multiple cortical sites will produce cognitive dysfunctions that were not present during standard SSS has been met.

### Bio-electrical accounts

In this feasibility study, we did not investigate the bio-electrical effect of MSS underlying the cognitive disruptions found. This effect can either be restricted to the two or three discrete stimulated sites or include tissue between these regions, creating a more distributed cortical effect. The current study cannot resolve between the two options, and arguments can be provided in both directions. On one hand, the spread of bipolar cortical stimulation has been previously shown to be limited to the circumferential range of 5mm radius [10], while MSS sites were farther apart (at least 2 cm), and in some cases in two distinct lobes (cases 8, 13 and 14). Moreover, when applying bipolar, biphasic stimulation with the ECOG strip, the onset-anode and onset-cathode electrode within each pair were randomly selected (randomly selected for each electrode pair, but constant across multiple stimulations of that pair). In this situation, the two stimulation locations are expected to repel electrical currents instead of attracting them. Additionally, in eight patients a cognitive effect was observed using the cortical strip electrode and the Ojemann stimulations. These two stimulating tools were manually operated by two separate personnel and thus could not have been phase locked. On the other hand, the spread of electrical field caused by an external stimulator and its ability to excite or inhibit neural populations relies on multiple, partly unknown factors, such as the cortical anatomy causing inhomogeneous tissue resistance, neuronal morphology and bio-electrical characteristics. This is particularly true in the cases where MSS may have been in phase and thus result in a complex quadropole (in case of double-site stimulation) or hexapole (in case of triple-site stimulation)[18,19]. Future studies should evaluate these possibilities using computational and animal research. Nevertheless, the results of this study suggest that functional criticality is not only based on specific local "hot spots" but may also involve more distributed and diverse cortical structures.

### Mechanistic accounts

If MSS caused stimulation of two or three restricted sites, several possible mechanisms could underlie the subsequent functional effect. One possibility is that these two nodes were both connected (structurally) to a third location and their simultaneous stimulation caused the disruption of this third location's function (while one of these two inputs was sufficient). A second possibility is that these nodes were directly connected by white matter (such as U-fibers) and that this connection is part of a network that serves a specific function (such as phonology or syntax, in the case of language). Yet a third possibility is that each of these nodes is part of a distinct network, each serving a different sub-function, and the co-activation of these networks is responsible for a more general function. For example, according to Indefrey & Levelt [20] phonological word production is composed of a lexical to phonological coding process and a syllabification process. If each of these processes is mediated by a specific cortical network, it may be possible that a small disruption in one will not cause an effect, but a simultaneous stimulation of both networks will result in phonological difficulties. While this initial study cannot answer these mechanistic questions, it provides some clues. First, there were cases where two-site stimulation caused an effect which qualitatively changed when adding a simultaneous stimulation in a third location. For example, patient 1 showed a shift from semantic to phonological effects, while patient 2 showed a reverse effect. Patient 8 showed a shift from syntactic disruption to semantic disruption. The qualitative nature of the changes in functional impairments seem to imply that language processing is based on a complex arrangement of multiple networks and their interaction rather than a serial cascade of phases as was often suggested in the original language models. This fits well with recent theoretical and experimental data showing that functional neural networks are often organized in small-world architecture [11,21,22]. Moreover, this result seems to imply that the same region can be part of multiple sub-networks serving different sub-functions as has been previously observed [12,23]. An important contribution to the understanding of this phenomenon, both mechanistically and clinically, will be pre- and post- operative comprehensive neuropsychological assessments. These may provide insights regarding the specific relation between the functional effects identified by each stimulation approach (SSS vs. MSS; two vs. three sites) and the resulting cognitive effect. Another way to increase our mechanistic understanding of MSS and its impact could be to combine MSS with positive SSS sites. In this preliminary study we were interested in the feasibility of multi-site stimulation and its ability to track functional regions which were silent in the standard single stimulation. Since cortical mapping during awake craniotomy is primarily aimed at preservation of patients’ functionality, when a functional site was found using SSS it was spared during resection, regardless of MSS attempts.

### Language functioning theoretical accounts

Language dysfunctions caused by MSS varied between patients and tumor locations and included primary production and comprehension dysfunctions; as well as secondary language dysfunctions, such as phonological and semantic paraphasias. These results suggest an additive role for MSS stimulation in mapping both primary and secondary language functions. The initial small group of patients reported here does not allow drawing conclusions regarding specific aspects of language processing. However, some thoughts regarding language functional organization may be derived. For example, recent studies using network fMRI and DTI, have distinguished between a ventral semantic network and a dorsal phonological network [24]. The phonological network includes the STG and premotor cortex. The semantic network includes more inferior temporal areas which are connected to IFG and MFG. Interestingly, the initial results presented here are relatively compatible with these findings, as the phonological effects we found were located in more posterior frontal regions compared to semantic effects (see Fig 2). In a different study, Xiang et al., [25] used connectivity fMRI to map the different networks of the IFG (Broca's region) using three seeds; Based on previous work [26,27] they relate these three networks to a phonological network (originating from pars opercularis), a syntactic network (originating from pars triangularis) and a semantic network (originating from pars orbitalis). These results are fairly compatible with the results of Saur at al., [24], and with our initial results, showing an anterior to posterior axis of IFG-MFG and an inferior to superior axis in the temporal lobe representing a semantic to phonological gradient. They also extend this axis and add a midway syntactic network. Interestingly, in accordance with this model, the single syntactic effect observed in our study, was observed during a simultaneous stimulation of the pars triangularis and the cortex just adjacent to the superior temporal sulcus. The location of the syntactic effect is compatible with previous studies showing the significance of the temporal pole to syntax processing[12]. The fact that only a single syntax effect occurred in 15 patients can possibly be explained by the complexity of this process[28], thus requiring a complicated and fine disturbance of activity and connectivity to disrupt this process. This may explain why syntactic effects are less frequent than semantic or phonological effects using standard DCS [29]. Altogether, syntax may be a good example of a linguistic process that can largely benefit from MSS.

MSS locations were found to partly converge with language fMRI activation. For example, for five of seven patients with a positive two-site MSS effect which could be localized, at least one of the two sites converged with the fMRI map of at least one of tasks. This seems to be at the range of previously reported sensitivity of fMRI as verified by SSS [30]. However, the small number of patients and variations in brain lesions and edema does not allow for a systematic comparison between fMRI and MSS. Particularly, since there were multiple types of MSS effects, it was not possible to correlate specific fMRI tasks to a specific stimulation effect. Future studies, with larger cohorts, should evaluate whether, for example speech arrest found with MSS correlates with production and not comprehension task related fMRI activations.

### Study limitations

This feasibility study harbors several limitations. First, while we saw no functional decline following resection (which spared both SSS and MSS positive regions), the clinical significance of MSS was not thoroughly assessed due to the small study cohort. To show that MSS is essential to functional preservation one should compare patients’ surgical outcome (in terms of functional status and EOR) to a matched-control group undergoing SSS alone under the same conditions. Future studies should aim for such a controlled setting, combined with thorough assessment of patients’ cognitive status both pre- and post- operatively in order to learn about the specific additive value of MSS. Second, as mentioned above, we did not provide a precise mechanistic explanation to the bio-electrical effect of MSS. MSS implementation as part of the routine intraoperative clinical practice relies on a better understanding of whether the intermittent (between stimulation sites) cortex is also affected. Our examination showed that MSS can be caused by different polarities and phase shifts across stimulation pairs, suggesting that in at least some of the MSS effects the tissue between sites was not affected, However, future studies should examine both polarity and phase changes in the same stimulation locations to reach more concrete conclusions. In-vitro MSS and concurrent recordings can also assist in elucidating the underlying mechanism. Moreover, most of the MSS attempts were performed on a linear ECoG strip. In the future, replacing the strip with a grid can allow more flexibility and thus a better understanding of the significance of current directionality and distance on the functional effects. Additionally, we did not evaluate MSS on sites with a positive SSS effect. Future studies should examine the effect of adding a second stimulation (in different locations) to positive SSS sites, and the effect of simultaneously stimulating two positive SSS sites. Computational studies attempting to model the effect of different stimulation parameters on current distribution caused by MSS are also called for. Finally, using imaging techniques (such as network fMRI and DTI) could provide additional insights to the nature of functional disturbance caused by MSS and its relation to known functional networks organization.

## Conclusion

Standard DCS allows the evaluation of the extent to which specific localized brain areas are necessary for the brain to achieve adequate cognitive functioning. In this work we observed a phenomenon in which locations where DCS did not elicit a functional disruption were still involved in critical aspects of language processing, possibly as part of a wider, more complex cortical system as observed using MSS. While the electrical and biological effects of MSS are still not clear, these results suggest that more distributed cortical activity may also have a critical role in cognitive processing, as suggested, but not proven by neuroimaging studies. We thus suspect that in the future, MSS could have a significant role in clinical preservation of cognitive function, as well as in a deeper understanding of the neural dynamics involved in cognitive processing.

## Supporting information

S1 Fig Example fMRI and MSS convergence. fMRI BOLD maps of the auditory definition task (Yellow, p<0.001, cluster size>70) and the visual definition task (Blue, p<0.01, cluster size>100) of patient 15 (color represents T-score). Conjunction of the auditory and visual tasks are marked in pink. MSS locations are marked in green. A and B represent locations of the stimulated ECoG pairs which did not produce an effect; and indeed A is not located where fMRI showed language related activations. C represents the location of the Ojemann stimulator, the stimulation of which, along with A and B cause a three-site MSS phonological effect; possibly relayed on regions indicated in fMRI activations corresponding to the location of B and C. The intra-operative photography including marked sites of effects is provided at the bottom right. s = superior, i = inferior, a = anterior, p = posterior.
(TIF)
