# Supplementary figures and images for: Intra-operative multi-site stimulation: Expanding methodology for cortical brain mapping of language functions
Source: PLoS One. 2017 Jul 10;12(7):e0180740. doi: 10.1371/journal.pone.0180740 (PMC5507257; doi:10.1371/journal.pone.0180740)

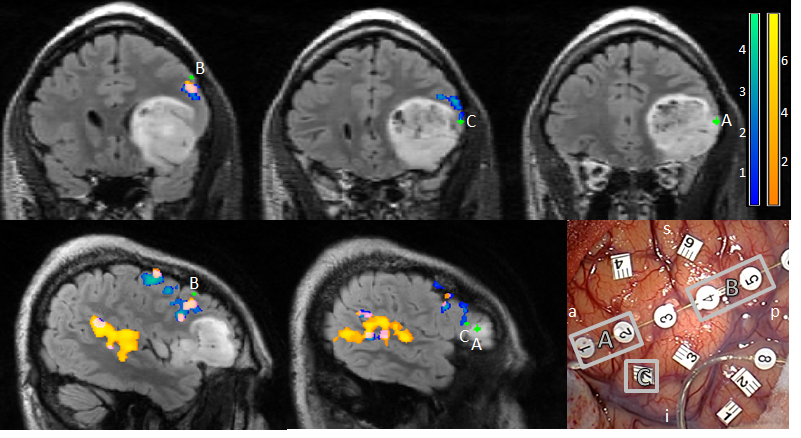

Supplement: S1 Fig — fMRI BOLD maps of the auditory definition task (Yellow, p<0.001, cluster size>70) and the visual definition task (Blue, p<0.01, cluster size>100) of patient 15 (color represents T-score). Conjunction of the auditory and visual tasks are marked in pink. MSS locations are marked in green. A and B represent locations of the stimulated ECoG pairs which did not produce an effect; and indeed A is not located where fMRI showed language related activations. C represents the location of the Ojemann stimulator, the stimulation of which, along with A and B cause a three-site MSS phonological effect; possibly relayed on regions indicated in fMRI activations corresponding to the location of B and C. The intra-operative photography including marked sites of effects is provided at the bottom right. s = superior, i = inferior, a = anterior, p = posterior. (TIF) [file pone.0180740.s001.tif]
